# Comparison between biliary plastic stents with and without application of silver nanoparticles: an *in-vitro* study of the biofilm formation

**DOI:** 10.1590/acb402825

**Published:** 2025-03-31

**Authors:** Victor Kalil Flumignan, Marcelo de Palma Sircili, Marcia Regina Franzolin, Ana Marisa Chudzinski Tavassi, Lígia Garcia Germano, Ana Vitória dos Santos Souza, Nicole Fernandes Silva, Newton Kiyoshi Fukumasu, Raphaela Marques dos Anjos, Jose Pinhata Otoch, Everson Luiz de Almeida Artifon

**Affiliations:** 1 Universidade de São Paulo – Faculty of Medicine – Postgraduate Program in Anesthesiology and Surgical Sciences – São Paulo (SP) – Brazil.; 2 Universidade de São Paulo – Butantan Institute and Interunit Graduate Program in Biotechnology – Laboratory of Genetics – São Paulo (SP) – Brazil.; 3 Universidade de São Paulo – Butantan Institute and Bacteriology Laboratory – São Paulo (SP) – Brazil.; 4 Universidade de São Paulo – Butantan Institute and Center for Development and Innovation – São Paulo (SP) – Brazil.; 5 Universidade de São Paulo – Institute for Technological Research – São Paulo (SP) – Brazil.; 6 Universidade de São Paulo – Medical School – São Paulo (SP) – Brazil.

**Keywords:** Biliary Tract, Biofilms, Cholangiopancreatography, Endoscopic Retrograde, Nanoparticles

## Abstract

**Purpose:**

Plastic biliary stents are a cost-effective treatment for biliary obstruction. Unfortunately, they have low patency, related to intraluminal biofilm formation. Silver nanoparticles (AgNPs) have been increasingly used in biomedicine because of its antibacterial properties. This study aimed to compare biofilm formation on stents with and without silver nanoparticle coatings when in contact with different bacterial culture medium.

**Methods:**

Different types of silver coatings were tested on plastic biliary stents. Two groups of stents were analyzed: one group with various types of silver nanoparticle coatings, and a negative control group with no coating. The stents were placed in different bacterial culture media and assessed for biofilm formation. Analysis was performed using confocal microscopy and direct colony-forming unit (CFU/cm^2^).

**Results:**

Quantitative analysis showed promising results with C16 coating, as *Escherichia coli* ATCC and *Pseudomonas aeruginosa* ATCC exhibited reduced growth in the AgNP-coated group (*p* < 0.05). However, when mixed samples, including clinical strains and *Staphylococcus aureus*, were tested, the AgNP coating did not inhibit bacterial growth.

**Conclusion:**

AgNP-coated stents are effective against certain strains, such as *E. coli* ATCC and *P. aeruginosa*. Further research is needed to explore potential improvements in the coating mechanism.

## Introduction

Endoscopic approaches are consolidated as the standard treatment for obstructive jaundice. Through endoscopic retrograde cholangiopancreatography (ERCP), with biliary stent placement, benign and malignant disease can be relieved^1^^–^ 4.

Despite being very effective and accessible, plastic stents have a medium patency of three months, when they usually get occluded, causing jaundice e sometimes cholangitis, being necessary a new ERCP, to exchange the stent. The precocious occlusion and the necessity of reapproaches lead to high costs to the health care and high morbidity to the patient.

The process of occlusion begins with the formation of bacterial biofilm in the surface of the stent. The presence of biofilm initiates an accumulation of sludge, playing a major role in stent’s occlusion. Biofilm is defined as a matrix comprising proteins, polysaccharides, nucleic acids, and lipids in differing quantities^5^.

Biliary sludge, in turn, is composed by cholesterol crystals, calcium bilirubinate and palmitate, bacteria and/or fungi, microbial byproducts, proteins, dietary fibers, and glycoproteins^1^^,^ 6.

Silver nanoparticles (AgNPs) are known for its bactericidal effect, without the high toxicity of silver ions, and have been used with good results in medical e pharmaceutical areas^7^. Reports affirm that AgNPs are effective in killing both gram-positive e gram-negative agents^8^^,^ 9. It was shown that the nanosize of the particles has a fundamental role in antibacterial action, since it improves the permeability of Ag ions on the cell. Also, the antibacterial action depends on other morphological characteristics such as surface and shape^10^. It was demonstrated the bactericidal activity of AgNPs when in contact with gram-negative bacteria (*Escherichia coli*), due to the increase in permeability, results in death of the cell^11^.

Regarding toxicity, analysis revealed that AgNPs are between 6,224 to 7,665 times less toxic than ion silver, when using *E. coli* as the microorganism model^12^. It is known that the toxicity of AgNPs depends on their size, shape and concentration^10^.

The aim of this study was to analyze if the use of AgNP is capable of preventing the formation of biofilms in biliary plastic stents.

## Methods

### Preparation of the substrate

Two groups of plastic biliary stents with and without AgNPs coating were analyzed. Polymethylmethacrylate (PMMA) discs of 5 × 2 mm, simulating plastic biliary stents, were analyzed. After sterilization with ultrasonic bath, the subtract were positioned 11 cm away from the target, for deposition of the film, using a pulsed direct current magnetron sputtering (pDCMS) technique. The Ag and C targets were activated, exposing the substrate to intermittent contact to Ag and C, resulting in an Ag-C coatings on plastic substrates. The AgNP film was deposited over an argon atmosphere of argon (Ar) of 3x10^-1^ Pa, for 10 minutes, power of 50 W for the Ag target and 300 W for the C target.

### Silver impregnation

Fragments designated as C1 underwent deposition composed solely of AgNPs. In an attempt to achieve more efficient inhibition of bacterial growth, the deposition type of C1 was modified to create a thicker silver-only film (similar to C1, but with greater thickness). This type of deposition, containing only thicker silver, was named C2.

In C15, carbon was associated with silver. Initially, a formulation of deposition involved a thick layer of AgNPs associated with carbon, with a higher quantity of silver than carbon. However, a formulation with less silver (approximately three times fewer AgNPs) associated with the presence of carbon, albeit with silver predominance in its composition, was also developed and named C16.

The C16 deposition type was further modified to include a higher quantity of carbon than silver in its composition, and it was named C17.

Furthermore, fragment C15 (thick silver layer–approximately three times thicker than C16) was modified with a predominant composition of carbon instead of silver. This type of deposition was designated C18 (Table 1).

**Table 1:** Types of deposition.

Fragment	Description
Negative control	Uncoated fragment placed in bacteria-free medium.
C0	Uncoated fragment of silver or carbon nanoparticles.
C1	Standard fragment, initially with silver nanoparticle deposition.
C2	Modification of fragment C1: deposition of silver nanoparticles with greater thickness than the original.
C15	Deposition containing silver nanoparticles and carbon, with silver predominance.
C16	Modification of C15 (three times fewer than silver nanoparticles).
C17	Type of deposition C16, but with predominance of carbon in its composition.
C18	Type of deposition C15, but with predominance of carbon in its composition.

Source: Elaborated by the authors.

### Bacteria

Bacteria standardized by the National Committee for Clinical Laboratory Standards (NCCLS) were analyzed: *E. coli* ATCC 25922, *Pseudomonas aeruginosa* ATCC 27853, *Staphylococcus aureus* ATCC 25923, Enteroaggregative *E. coli* 042 (serotype O44:H18)^13^, and Atypical Enteropathogenic *E. coli* BA 4157 (serotype ONT:H25).

### Biofilm formation on abiotic surfaces: crystal violet

The quantitative analysis of biofilm formation on polystyrene plates was conducted following the methodology described by Christensen^14^ and Sheikh^15^. For biofilm formation, samples were cultured in overnight pre-inoculum at Luria Bertani (LB) medium under agitation. After growth, the pre-inoculum was diluted in the culture medium to be tested in 96-well polystyrene plates. The plate was then incubated for 24 hours at 37°C. For biofilm quantification, the bacterial growth medium was removed, and the wells were washed three times with a phosphate buffered saline (PBS) solution to remove planktonic bacteria. After washing, the bacteria were fixed with ethanol and with 0.5% crystal violet solution for 5 minutes. Subsequently, the dye was removed, and the wells were washed four times with PBS to remove the excess dye.

**Figure 1:**
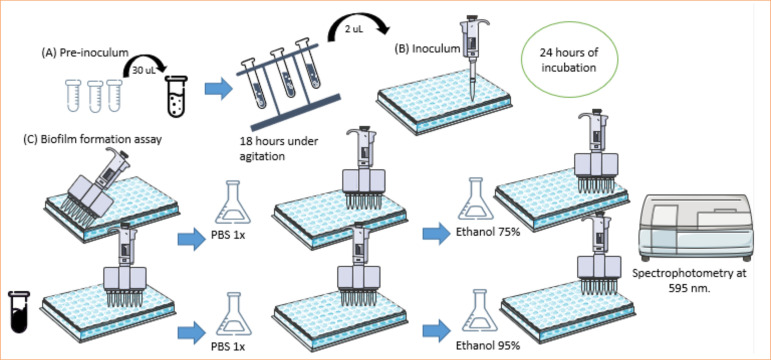
Biofilm formation assay with crystal violet.

Source: Elaborated by the authors.

After washing, the plate was left at room temperature until completely dried. Then, 95% ethanol was added for 2 minutes to solubilize the biomass. The absorbance was measured using an enzyme linked immunosorbent assay (ELISA) reader Multiskan EX (Thermo Fisher Scientific, United States of America) at the wavelength of 595 nm (Fig. 1).

### Biofilm formation test on abiotic surface: scanning electron microscopy analysis

For the biofilm formation test using scanning electron microscopy analysis, after a 72-hour incubation period of bacteria on PMMA discs, each well containing a disc was washed three times with PBS and then fixed with Karnowsky for 2 hours. The bacteria were washed three times for 15 minutes each with 0.1 M sodium cacodylate buffer, pH 7.2.

After fixation the material concentrated on the Karnowsky-fixed disc, it was pipetted and left for 20 minutes. Excess material was removed, followed by three washes.

The discs were dehydrated successively with ethanol. The dehydrated materials were dried using the critical point drying method and subsequently coated with a thin layer of gold. Fragments were analyzed under high vacuum using a FEI Quanta 250 scanning electron microscope.

### Biofilm formation test on abiotic surface: confocal microscopy analysis

For tests conducted using confocal microscopy analysis, after the incubation period, each well of the plate was washed with PBS. Subsequently, bacteria were fixed with 4% paraformaldehyde in PBS. After additional washes, propidium iodide (Molecular Probes, United States of America) was added at the final concentration of 1:1,000. Coverslips were then incubated for 45 minutes in the dark. The slides were observed using a fluorescence confocal microscope at a magnification of 630x, using an LSM 510 Meta microscope (Zeiss, Germany). A 570-719-nm interference pass (IP) filter and a 543-nm wavelength (excitation) were used. LSM Image Browser software was employed for sample capture and analysis.

### Statistical analysis

The number of bacteria that adhered to the prosthesis was transformed into base 10 logarithms and expressed as log10 colony forming units (CFU)/cm^2^/day ± standard deviation. All data were subsequently analyzed using specific statistical methods.

Experiments were conducted in triplicate with three repetitions each, analyzed by analysis of variance (Excel). For example, three uncoated prostheses and three coated stents were averaged, and statistical results were analyzed (standard deviation).

## Results

### Biofilm formation analysis

For biofilm formation analysis, fragments with and without AgNp coating were placed in culture medium containing different bacteria. Tests with crystal violet were performed.

Stent fragments were analyzed with various AgNPs coatings to determine the optimal formulation. Analyses were consistently conducted in comparison to a negative control (fragments placed in LB medium without bacteria) and with fragments both coated and uncoated with silver, the latter always labeled as C0. The other fragments were designated as C2, C15, C16, C17, and C18.

**Figure 2:**
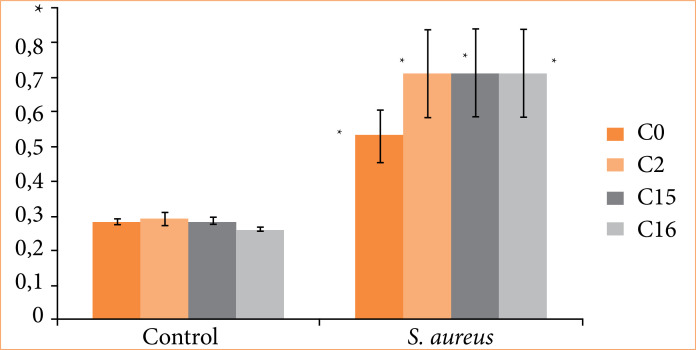
Biofilm formation test at 37°C for 24 hours, analyzed by crystal violet on different surfaces. Polymer surfaces without coating and coated with silver nanoparticles were tested: negative control and *Staphylococcus aureus* samples.

Source: Elaborated by the authors.

When cultured in media containing *S. aureus*, the coatings C2, C15, and C16 showed no protective effect; that is, none of them exhibited inhibition of growth compared to the C0 fragment (Fig. 2).

**Figure 3:**
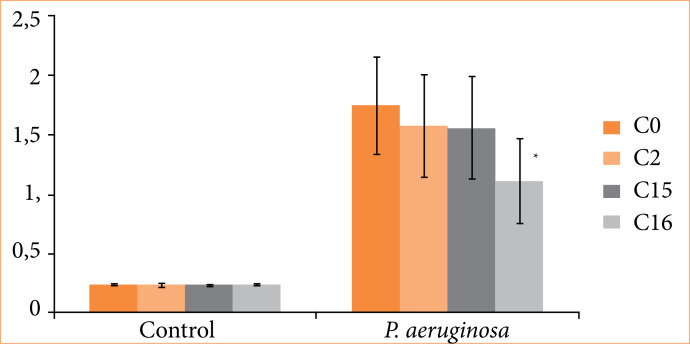
Biofilm formation test at 37°C for 24 hours, analyzed by crystal violet on different surfaces. Polymer surfaces without coating and coated with silver nanoparticles were tested: negative control and *Pseudomonas aeruginosa*.

Source: Elaborated by the authors.

When placed in a culture medium containing *P. aeruginosa*, all coatings (C2, C15, C16) showed reduced bacterial growth compared to C0. However, statistical significance (*p* < 0.05) was observed only for C16 (Fig. 3).

**Figure 4:**
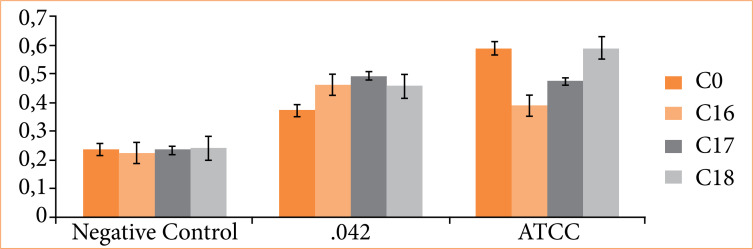
Biofilm formation test at 37°C for 24 hours, analyzed by crystal violet on different surfaces. Uncoated and silver nanoparticle-coated plastic biliary stents were tested. Sample 042, prototype samples of diarrheagenic *Escherichia coli* of the enteroaggregative *E. coli* (EAEC) pathotype, and ATCC *E. coli* 25922 sample.

Source: Elaborated by the authors.

When compared to sample C0, all samples (C16, C17, and C18) showed higher bacterial biofilm in media containing *E. coli* 042, although statistical significance (*p* < 0.05) was observed only for C17. Similarly, when compared in media containing E. coli ATCC 25922 (commensal strain), all samples (C16, C17, and C18) exhibited lower bacterial growth than C0, with statistical significance (*p* < 0.05) observed only for C16 (Fig. 4).

**Figure 5:**
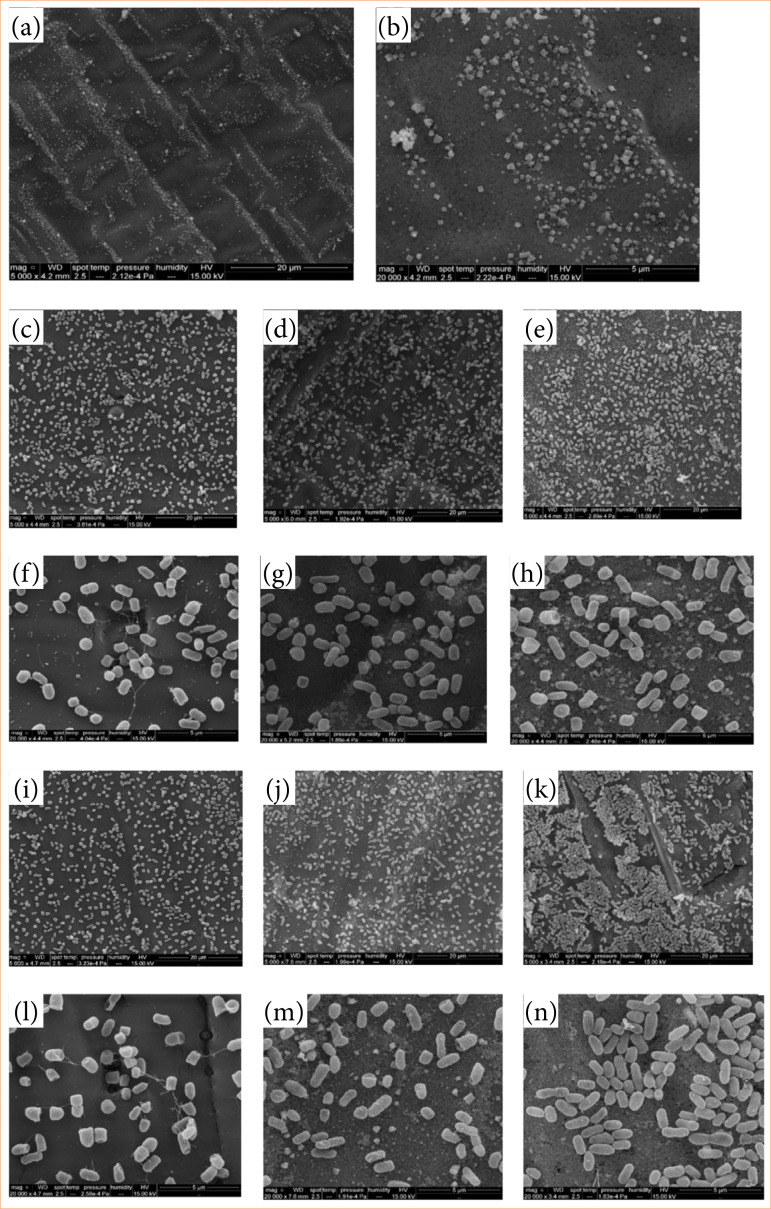
Scanning electron microscopy images: **(a and b)** Fragment C16: Polymer coated with silver nanoparticles [**(a)** 5,000x magnification, **(b)** 20,000x magnification]. (c–e) Biofilm formation of diarrheagenic *Escherichia coli* Ba4157, pathotype atypical enteropathogenic E. coli (aEPEC), on polymer coated with silver nanoparticles [**(a)** C0, **(b)** C16, **(c)** C18–magnification 5,000x. (f–h) Biofilm formation of diarrheagenic *E. coli* Ba4157, pathotype atypical enteropathogenic *E. coli* (aEPEC), on polymer coated with silver nanoparticles: [**(a)** C0, **(b)** C16, **(c)** C18–magnification 20,000x]. (j–k) Biofilm formation of E. coli ATCC samples on polymer coated with silver nanoparticles [**(a)** C0, **(b)** C16, **(c)** C18–magnification 5,000x. (l–n) Biofilm formation of E. coli ATCC samples on polymer coated with silver nanoparticles [**(a)** C0, **(b)** C16, **(c)** C18–magnification 20,000x.

Source: Elaborated by the authors.

Electron microscopy images illustrated the results of bacterial biofilm formation. The images showed fragments without AgNPs deposition (C0), and fragments coated with C16 and C18, prior to contact with the culture medium. Subsequently, after exposure to culture medium containing *E. coli* BA4157 (diarrheagenic type), electron microscopy photographs (at 5,000x magnification) demonstrated reduced bacterial growth on the C16 coating compared to C18 and the uncoated silver nanoparticle fragment (C0). The images provided detailed observations at 20,000x magnification. Similarly, electron microscopy images taken after exposure to culture medium containing *E. coli* ATCC also showed reduced bacterial growth on the C16 sample compared to C18 and C0, observed at 5,000x magnification, and further detailed at 20,000x magnification (Fig. 5).

**Figure 6:**
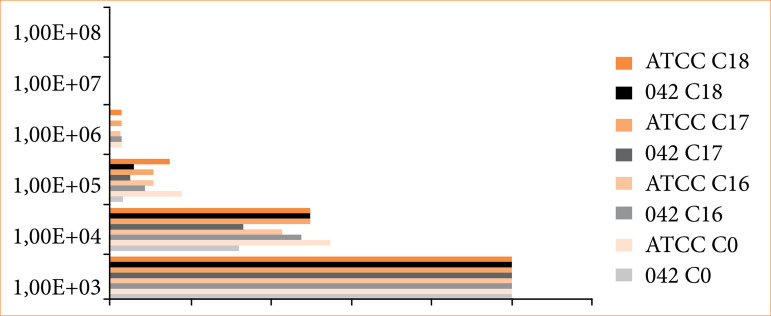
Comparative analysis of colony-forming unit (CFU) counts from samples contained in biofilms obtained from different coating types.

Source: Elaborated by the authors.

In Fig. 6, a partial comparative analysis of different coating types with various tested bacteria is presented. Coatings C0, C16, C17, and C18 are compared when in contact with *E. coli* ATCC and *E. coli* 042. Initially, at the extremes of the graphs, it can be observed that all coating types exhibit equal growth up to 10^3^, and nearly all samples show minimal and equivalent growth up to 10^6^, except for C18 and C17 samples when in contact with the pathogenic strain *E. coli* 042 (diarrheagenic), which did not exhibit growth at this level. In the middle portion of the graph, significant differences are noted in growth up to 10^4^ and 10^5^. As previously described and evident in this graph, the superiority of the C16 coating over others is observed when evaluating ATCC samples, a result not confirmed when testing clinical samples such as E. coli 042.

## Discussion

The modification of surfaces of polymers used in biomedicine such as polytetrafluoroethylene, polyethylene, and polyurethane can alter their biocompatibility, as well as cell proliferation and differentiation^16^. The use of AgNPs on these surfaces, in association with other elements, has shown progress in bacterial control over the past years. It is believed that the biocidal mechanism of AgNPs is due to the release of silver ions, direct contact between the AgNPs and bacteria, and the generation of free radicals^13^^,^ 17. The association of gold, silver, carbon, titanium, among others, is common, which led us to believe that their use can be beneficial when used in plastic biliary stents (usually composed of polyurethane or polyethylene). However, there is still little evidence regarding the use of AgNPs in biliary stents, and therefore scarce development of materials in this area. The methodologies for these studies are limited and used only for antimicrobial activity analysis. Studies focused on biofilm inhibition still show deficient methodologies. This study focused on biofilm formation inhibition, and not on antimicrobial activity.

In addition, after the development of the materials, it is still difficult to find qualified professionals for conducting microbiological analyses, especially regarding biofilm analyses, as well as places with technology and qualified professionals for the development and application of silver and carbon nanoparticles in polymers.

Various types of deposition were tested, some composed solely of AgNPs (with different film thicknesses), and others containing carbon combined with silver (also with different thicknesses). Initially, depositions containing only AgNPs were tested, not yielding effective results as expected. Even after increasing the thickness and amount of silver in the deposition, the results were not effective. An association, increasingly used in biomedicine, composed of silver and carbon nanoparticles (in different models, with a predominance of silver or carbon in their composition) was then the chosen option. Initially, the results were not positive, as most tests did not show inhibition of growth compared to the controls. A film was then developed with approximately three times less silver, maintaining the association with carbon (still with a predominance of silver over carbon), yielding positive results, showing bacterial growth inhibition. Attempts were also made to associate previous models, with a predominance of carbon instead of silver in their composition. This association was not efficient in inhibiting bacterial growth when placed in culture media with the different tested bacteria.

It was initially observed that deposition types C2, C15, and C16 did not show the ability to prevent the growth of *S. aureus*, a fact that cannot yet be fully understood. The hypothesis generated so far is that bacteria have different types of surfaces and react differently when in contact with silver. The presence of fimbriae in some bacteria could, for example, lead to adhesion to AgNPs, leading to biofilm formation and bacterial growth. To confirm the hypothesis and understand why the deposition types are efficient for some bacteria and not for others, future studies are necessary.

It could be also observed that the association between silver and carbon nanoparticles was efficient and showed good results in inhibiting bacterial growth. Its superiority was demonstrated when the film is composed of a formulation between silver and carbon, in which carbon improves the interaction of silver with the bacteria. This successful relationship was demonstrated in previous studies, showing that the union of carbon and silver forms a type of material that facilitates the interaction of silver with bacteria, with silver and carbon performing worse in terms of bactericidal or bacteriostatic effect when not combined^18^^,^ 19. In these cases, the coating surface is composed only of silver. In addition, the most efficient formulation occurred when the film composition was predominantly silver over carbon.

However, when tested with *P. aeruginosa*, deposition types C2, C15, and C16 showed bacterial growth inhibition, demonstrating a protective effect for this bacterium, but only with statistical significance in C16. It was understood that this type of coating was superior to the others, and new analyses were then scheduled with C16 and new formulations, based on the C16 coating type.

Deposition type C16 was compared with new formulations: C17 and C18, all in media containing *E. coli* ATCC 25922 (commensal). Bacterial growth inhibition was again observed in all deposition types, but only with statistical significance in C16, once again demonstrating that the deposition of AgNPs in a thinner layer, associated with carbon in its formulation, and with a surface composed solely of silver, is the most efficient so far. C16, C17, and C18 were also tested in media containing *E. coli* 042 (diarrheagenic), with no bacterial growth inhibition observed in any of the samples, including C16. This fact cannot be precisely explained, but, once again, it emphasizes the different surfaces of bacteria and, therefore, the different interactions with coating surfaces. It was demonstrated that AgNPs bactericide effect depends on the concentration of silver, and that this concentration is different depending on the bacteria. In this study, *P. aeruginosa* needed higher concentrations of AgNPs than *E. coli*, to prevent bacterial growth^20^.

When compared to the literature, the results seem negative, but it is important to note that most previous studies used ATCC samples, achieving good results, as in this study; when these samples were tested, positive results were obtained. However, the studies did not use clinical samples, as the present study did. In addition to using pathogenic samples, samples from obstructed stents were used, thus testing the deposition types with the bacteria present in patients with obstructed stents. In both situations, no growth or biofilm formation inhibition were observed. Concern arises about the efficiency of these films when used in clinical practice.

In any case, extensive studies with different types of deposition, and with the presence of ATCC and clinical samples, are necessary to better understand the interaction between bacteria and AgNPs.

By the use of silver and carbon nanoparticles, bactericidal and bacteriostatic action in ATCC samples was observed, data not confirmed when clinical bacteria were analyzed, suggesting that more studies containing both ATCC and clinical samples are necessary, as well as improvements in the deposition method of these stents. The possibility of developing types of stents with different shapes that do not favor biofilm formation in their distal portion should be also considered. In summary, new strategies are necessary, since plastic stents remain the most accessible nowadays.

## Conclusion

Stents coated with silver and carbon nanoparticles demonstrated partial effectiveness in inhibiting the growth of bacterial species.

## Data Availability

The data will be available upon request.
